# Influence of Obesity on Heart Rate Variability in Nurses with Age and Shift Type as Moderators

**DOI:** 10.1155/2021/8119929

**Published:** 2021-11-17

**Authors:** Wen-Pei Chang, Chia-Hui Wang, Yen-Kuang Lin

**Affiliations:** ^1^Department of Nursing, Shuang Ho Hospital, Taipei Medical University, New Taipei City, Taiwan; ^2^School of Nursing, College of Nursing, Taipei Medical University, Taipei, Taiwan; ^3^Graduate Institute of Athletics and Coaching Science, National Taiwan Sport University, Taoyuan, Taiwan

## Abstract

Obesity is a risk factor of cardiovascular disease-related mortality and may be associated with changes in the autonomic nerve activity. Nurses working shifts and caring for patients are under great mental and physical pressure, and research has proven that these can negatively affect the body. The objective of this study was to examine the influence of obesity in nurses on their heart rate variability (HRV) and determine whether age or shift type moderates this influence. A questionnaire survey and HRV measurements were conducted on nurses at a hospital in Taiwan during a routine employee health checkup. HRV analysis was conducted using a noninvasive HRV monitor for five minutes. A total of 242 nurses with a mean age of 28.98 ± 6.56 years were enrolled in this study. An overly large waist circumference (WC) had a negative impact on high frequency (HF), low frequency (LF), and standard deviation of normal-to-normal interval (SDNN), while an overly high body mass index (BMI) had a negative impact on very low frequency (VLF) and SDNN. The interaction term “overly large WC × age” had a negative impact on HF (**β** = −0.21, **p** = 0.010) and LF (**β** = −0.18, **p** = 0.030), whereas the interaction term “overly high BMI×age” had a negative impact on HF (**β** = −0.27, **p** = 0.001), LF (**β** = −0.19, **p** = 0.023), and VLF (**β** = −0.17, **p** = 0.045). The interaction terms “overly large WC × shift type” and “overly high BMI × shift type” did not influence any HRV parameters. As age increased, so did the degree to which the HF and LF of nurses with an overly large WC were lower than normal, and so did the degree to which the HF, LF, and VLF of nurses with an overly high BMI were lower than normal.

## 1. Introduction

The autonomic nervous system (ANS) controls the activities of the organs, such as the heart. This system is divided into two parts with mutually inhibitory actions: the sympathetic nervous system and the parasympathetic nervous system. The sympathetic nervous system can accelerate the heart rate, dilate pupils, decrease gastrointestinal motility, cause perspiration, and strengthen muscles to deal with stressful or dangerous situations. Conversely, the parasympathetic nervous system can lower the heart rate, constrict pupils, increase gastrointestinal motility, reduce perspiration, and relax muscles so that the body can relax. The two systems inhibit each other to maintain a balance [1]. The ever-present influences of the sympathetic and parasympathetic nervous systems on heart rate are reflected in heart rate variability (HRV). In recent years, the influence of rotating shift work on HRV has been receiving a growing amount of attention. Studies comparing the HRV of individuals working and not working rotating shifts have found a greater likelihood of dysautonomia among the former. Individuals working rotating shifts are thus at greater risk of cardiac dysrhythmia, which can lead to sudden death [2, 3]. HRV has even been found to be a predictor of postmyocardial infarction mortality [4].

By using HRV to analyze the autonomic function of obese individuals, increases in adult obesity and decreases in HRV have been found to be associated with dysautonomia and result in ANS dysfunction [5]. Moreover, a significant correlation exists between ANS regulation and cardiovascular disease-related mortality. Such cardiovascular diseases include hypertension and hemorrhagic shock [6, 7].

Obesity refers to the excessive presence of adipose tissue in the body, and adipose tissue is now considered a complex organ [8]. Adipocytes can secrete vasoactive hormones and proteins collectively referred to as vasoactive factors, which include tumor necrosis factor *α*, interleukin 6, and leptin. These substances can affect metabolism and cardiovascular regulation [9]. Adipose tissue is also closely associated with obesity, diabetes, and other complications [10].

The ANS can regulate smooth muscle movement in human organs as well as the secretion of hormones from endocrine glands and is responsible for the release of neurotransmitters [11]. Catecholamines are a group of biogenic amine neurotransmitters, to which epinephrine and norepinephrine both belong. The former is associated with energy metabolism whereas the latter is connected to neurotransmission. Both are affected by sympathetic nerve activity. Imbalanced catecholamine concentrations can lead to dysautonomia in the sympathetic and parasympathetic nervous systems [12]. Obesity-associated induction of increased catecholamine secretion from the adrenal gland indicates a strong correlation between obesity and the ANS [13].

Panel surveys of the lifestyles of nurses have shown that stress from rotating shifts and caring for patients often causes fluctuations in their endocrine systems, which can in turn lead to obesity or chronic diseases [14]. In addition, rotating shifts can also affect the dietary habits of nurses, such as increasing food intake, thereby increasing the chances of suffering from metabolic syndromes such as obesity and diabetes [15]. Rotating shifts also disrupt the daily routines of nurses, change what they eat, and induce irregular meal times. In the long term, these changes affect their endocrine systems and often result in obesity [16].

**Figure 1:**
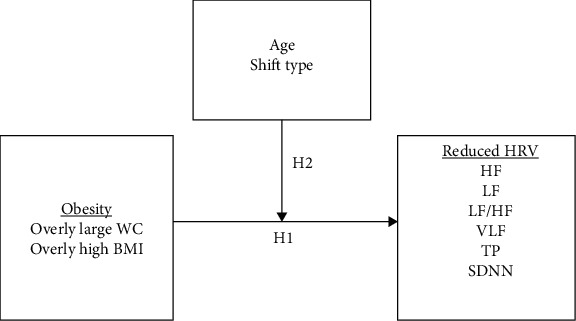
Research hypotheses and framework. Abbreviations: HRV: heart rate variability; HF: high frequency; LF: low frequency; LF/HF: low- to high-frequency power ratio; VLF: very low frequency; TP: total power; SDNN: standard deviation of normal-to-normal interval.

Despite literature indicating a close connection between obesity and HRV, little research has been done on the correlation between obesity and HRV in nurses working rotating shifts. Furthermore, the factors that influence HRV also include age, and research has shown that rates of obesity increase with age [17]. Thus, the objective of this study was to understand the influence of obesity on HRV in nurses.  Figure 1 displays the research framework, and our proposed hypotheses were as follows.

**Hypothesis 1.**

High waist circumference (WC) or body mass index (BMI) reduces HRV.

**Hypothesis 2.**

Age and work influence the impact of waist circumference (WC) or body mass index (BMI) on HRV.

## 2. Materials and Methods

### 2.1. Study Design and Sample

This study is a subproject of a large-scale project. We adopted a comparative-correlational design for this study. During routine annual health checkups for nurses at a teaching hospital in Taiwan from February to April 2018, we explained the purpose of our study to the nurses and gained their consent to collect their basic information and HRV parameters. In accordance with the Personal Data Protection Act, each participant was assigned an identification code to maintain anonymity. A total of 242 valid samples were obtained.

### 2.2. Research Instruments

A questionnaire and HRV analysis were the primary research instruments of this study. The content of the self-administered questionnaire included gender, age, shift type, medical history, and smoking and drinking habits of the nurses.

An increase in BMI, defined as body mass (kg)/body height^2^ (m^2^), indicates weight gain and obesity in the entire body. The participants of this study were divided into two groups: **BMI** < 24 kg/m^2^ was defined as normal weight or underweight, and**BMI** > 24 kg/m^2^ was defined as overweight or obese. Abdominal obesity was determined using WC; men with a WC over 90 cm and women with a WC over 80 cm were regarded as overly large [18, 19].

Nurses whose departments routinely require rotating shifts were considered “shift workers,” while those who did not need to rotate shifts and worked fixed day shifts were considered “fixed day shift workers.” All of the shift workers worked slowly rotating shifts, in which they worked the same shift for a month and then changed shifts the next month. There were three eight-hour shifts a day : day shift (08:00-16:00), evening shift (16:00-24:00), and night shift (24:00-08:00).

HRV analysis was conducted using a noninvasive HRV monitor (model: 8Z12, Cloud Care Inc.), which assesses the HRV for five minutes and then displays the individual's ECG signal on its liquid crystal display. ECG and HRV Analysis System v 0.3 beta were used to link the HRV monitor to a computer for HRV analysis. This involved photoplethysmography (PPG); the signals of which can be used to further understand peripheral blood circulation and provide a simpler, noninvasive way of measuring physiological signals. Based on the principle of light sensors absorbing light energy, the signals resulting from changes in light are recorded. When light penetrates tissues in the body, some of the light is absorbed and scattered by muscle tissue and blood. When the blood pumped from the heart reaches the blood vessels, the increase in blood causes slight changes in the volume of the peripheral capillaries. With LED light and light intensity detection, the signals following light absorption change as a function of variations in blood volume during heartbeats. This is the principle of PPG [20, 21].

During the two hours prior to the HRV analysis, nurses were confirmed to not have eaten; not have had any alcohol, tea, or caffeinated beverages; and not have engaged in strenuous activity. The safety and inspection procedures of the HRV monitor were explained before the analysis, which was conducted in a quiet separate room at room temperature in suitable lighting. The participants were asked to sit in a comfortable position on a chair with a backrest and were reminded to remain still, not talk, not close their eyes, and keep breathing naturally during the analysis. The HRV parameters in this study were as follows [22]:

1. Heart rate (HR): normal range between 60 and 80 beats per minute
2. High frequency (HF) (ms^2^): variance of the normal heart rate in the high-frequency range extracted using the frequency domain; this represents the activity of parasympathetic nerves
3. Low frequency (LF) (ms^2^): variance of the normal heart rate in the low-frequency range extracted using the frequency domain; this represents the activity of sympathetic and parasympathetic nerves
4. Low- to high-frequency power ratio (LF/HF): an indicator reflecting sympathovagal balance or sympathetic regulation
5. Very low frequency (VLF) (ms^2^): variance of the normal heart rate in the very low0frequency range extracted using the frequency domain
6. Total power (TP) (ms^2^): total variance of the normal heart rate in the high-frequency, low-frequency, and very low-frequency ranges extracted using the frequency domain; this clinically represents the overall HRV assessment
7. Standard deviation of normal-to-normal interval (SDNN) (ms): standard deviation of intervals between beats in time-domain heart rate data with a normal sinus rhythm; this is the most clinically recommended indicator of overall HRV in time-domain analyses. The optimal range of SDNN is 20-45; higher values indicate greater variability and stronger parasympathetic nerve activity, whereas lower values represent lower variability and weaker parasympathetic nerve activity

### 2.3. Statistical Analyses

Data processing was performed using SPSS 19 (SPSS Inc., Chicago, IL, USA). The descriptive analysis encompassed the demographic variables of the nurses and HRV analysis. Shift workers and fixed day shift workers were compared using chi-square tests and *t*-tests.

To determine the impact of obesity on HRV, we employed overly large WC and overly high BMI as independent variables and used multiple linear regression analysis to conduct impact analysis on the six HRV parameters (i.e., HF, LF, LF/HF, VLF, TP, and SDNN) with the enter method. Age and shift type were control variables.

We then used multiple linear regression analysis to conduct impact analysis on HRV parameters, with overly large WC or overly high BMI as the independent variable and age as the moderator. The moderating effects of the interaction effects between the two on HRV parameters were then examined. Significant interaction effects would indicate that age has a moderating effect on HRV. Similarly, we then performed multiple linear regression analysis using overly large WC or overly high BMI as the independent variable and shift type as the moderator, and the moderating effects of the interaction effects between the two on HRV parameters were examined.

## 3. Results

### 3.1. Demographic and HRV Distributions of Nurses

The study comprised a total of 242 nurses. Their mean age was 28.98 ± 6.56 years, and the vast majority was women (234 individuals, 96.7%). Most of the participants did not have a habit of smoking (230 individuals, 95.0%) or drinking (178 individuals, 73.6%), and most had no medical history of hypertension (241 individuals, 99.6%) or diabetes (239 individuals, 98.8%). Among the participants, 64 nurses (26.4%) worked fixed day shifts, whereas 178 nurses (73.6%) worked rotating shifts. In the demographic distributions of the two groups, only age presented a significant difference (*p* < .001); the mean age of those who worked fixed day shifts was 34.26 ± 8.18 years, which was significantly higher than the mean age of 27.09 ± 4.59 years of those who worked rotating shifts. The HRV parameters were found to have no significant relationship with shift type (Table 1).

### 3.2. Influence of Obesity on HRV

As shown in Table 2, the regression analysis of the influence of an overly large WC on HRV parameters revealed that the regression coefficient of the influence on HF was significantly negative (*β* = −0.14, *p* = 0.026), that of the influence on LF was significantly negative (*β* = −0.14, *p* = 0.035), and that of the influence on SDNN was significantly negative (*β* = −0.14, *p* = 0.029).

The regression analysis of the influence of an overly high BMI on HRV parameters revealed that the regression coefficient of the influence on VLF was significantly negative (*β* = −0.15, *p* = 0.023) and that of the influence on SDNN was significantly negative (*β* = −0.15, *p* = .020) (Table 2).

Note that *B* is the unstandardized regression coefficient, and *β* is the standardized regression coefficient.

### 3.3. Moderating Effects of Age on the Influence of Obesity on HRV

Table 3 shows the results of the regression analysis on the moderating effects of age on the influence of an overly large WC on the HRV parameters with the interaction term “overly large WC × age.” The regression coefficient of the influence on HF was significant (*β* = −0.21, *p* = .010) and that of the influence on LF was significant (*β* = −0.18, *p* = 0.030).

The regression analysis on the moderating effects of age on the influence of an overly high BMI on the HRV parameters revealed that with the interaction term “overly high BMI × age,” the regression coefficient of the influence on HF was significant (*β* = −0.27, *p* = 0.001), that of the influence on LF was significant (*β* = −0.19, *p* = 0.023), and that of the influence on VLF was significant (*β* = −0.17, *p* = 0.045) (Table 3).

### 3.4. Moderating Effects of Shift Type on the Influence of Obesity on HRV

Table 4 shows the results of the regression analysis on the moderating effects of shift type on the influence of WC on the HRV parameters with the interaction term “WC × shift type.” The regression coefficient of the influence on HF, LF, LF/HF, VLF, TP, and SDNN did not reach the level of significance.

The regression analysis on the moderating effects of shift type on the influence of BMI on the HRV parameters indicated that with the interaction term “BMI × shift type,” the regression coefficient of the influence on HF, LF, LF/HF, VLF, TP, and SDNN did not reach the level of significance (Table 4).

## 4. Discussion

The results of this study revealed that after age and shift type were controlled, nurses with an overly large WC had significantly lower HF, LF, and SDNN, while nurses with an overly high BMI had significantly lower VLF and SDNN. As age increased, so did the degree to which the HF and LF of nurses with an overly large WC were lower than normal, and so did the degree to which the HF, LF, and VLF of nurses with an overly high BMI were lower than normal. The differences between the HRV parameters of nurses with an overly large WC or an overly high BMI and those of nurses with normal or smaller/lower WC or BMI did not vary with whether the nurses worked rotating shifts or a fixed shift.

We employed PPG to measure various HRV parameters. PPG optically measures changes in blood flow volume in blood vessels. When LED light penetrates skin, tissues, and blood vessels, the amount of light reflected changes with the differences in vessel walls caused by heartbeats. The amount of light reflected varies with skin color as well as the wavelength of the light [21]. Because external light sources can easily affect photodiodes when they receive signals, it is important that the sensor is in tight contact with the skin. PPG and ECG signals can also be combined to calculate blood pressure, which has greatly increased the accuracy of obtaining blood pressure values [23].

In addition, research has indicated that linear methods are overly simple because physiological data (such as R-R intervals) that are input into linear analysis methods can only represent the linear ratios existing between the input and output signals; thus, the complex conditions of the heart rate cannot be fully represented [24]. The human system itself is a complex nonlinear system in which only limited data can be obtained from the observation of a single parameter alone. Moreover, self-similarity exists within the internal structures of this complex system, meaning that patterns on smaller scales may be replicated on large scales [25]. Small fluctuations could be missed when using large-scale measurements, and these small fluctuations may contain even smaller fluctuations. For this reason, no absolute value can be measured, no matter how small the measurement scale is. This phenomenon is called a fractal structure. Many similar fractal structures exist in the human system. The careful analysis of the rate of each heartbeat could uncover that the heart rate exhibits immense variability. This continuous HRV has been demonstrated to contain fractal structures. Such unique structures have a certain degree of complexity; the peak-to-peak intervals of each heartbeat vary, and analysis of the rate of each heartbeat reveals that the heartbeats are full of variability. Thus, the analysis and processing of physiological data that are input into nonlinear analysis methods do not maintain linear ratios with the output signal, which applies to the HRV analyzed in this study [26].

The ANS plays a crucial role in regulating energy consumption and body fat content; however, there is no consensus so far on whether this is different in obese individuals. Guarino et al. believed that sympathetic and parasympathetic nerve activities change [12], whereas Browning et al. believed that it is mainly the parasympathetic nerve activity that reduces [27]; Straznicky et al. though indicated that obesity is more associated with the reduction in the sympathetic nerve activity [28]. Following the control of age and shift type, the results of this study revealed that nurses with an overly large WC displayed significantly lower HF, which represents parasympathetic nerve activity, lower LF, which represents sympathetic and parasympathetic nerve activity, and lower SDNN, which represents overall autonomic nerve activity. Nurses with an overly high BMI displayed significantly lower VLF, which is associated with the sympathetic nerve activity, and lower SDNN, which is associated with the overall autonomic nerve activity. Thus, our findings indicate changes in both the sympathetic and parasympathetic nerve activities in obese or overweight nurses.

Research from as early as 1987 has demonstrated that like left ventricular ejection fraction (LVEF) or ventricular arrhythmia, SDNN can serve as an independent predictor of postmyocardial infarction mortality [29]. A later study investigated whether HRV could be used to predict sudden cardiac deaths in patients with congestive heart failure. Among 127 patients, those with lower SDNN (<65.3 ms) presented a higher sudden death rate. In addition, SDNN was the only HRV factor that could independently predict the overall mortality rate [30]. Following the control of age and shift type, the results of our study demonstrated that nurses with an overly large WC or an overly high BMI also had lower SDNN, thereby supporting that SDNN can serve as an effective predictor of cardiovascular diseases in nurses.

Regarding the influence of an overly large WC or overly high BMI on HRV parameters, Windham et al. performed a longitudinal study to analyze the connection between HRV and WC and found that increases in the WC of middle-aged individuals with a mean age of 45 years were associated with decreases in SDNN; thus, abdominal obesity is highly correlated with HRV [5]. Grewal and Gupta studied 100 volunteers who were recruited in India. The 50 participants with a BMI > 30 kg/m^2^ were designated as the study group (mean age 33.38 ± 8.73 years), and the other 50 participants with a BMI < 30 kg/m^2^ were the control group (mean age 34.86 ± 10.24 years). After six noninvasive ANS function tests, the authors discovered that the sympathetic and parasympathetic nerve functions of the study group were all lower than those of the control group, which means that an overly high BMI affects the sympathetic and parasympathetic nerve functions [31]. Our study demonstrated that the age of nurses strengthens the influence of an overly large WC on HF and LF as well as strengthens the influence of an overly high BMI on HF, LF, and VLF. Age influences the impact of obesity in nurses on the autonomic nerves in their cardiovascular system, and this impact is even more prominent in older nurses, which reduces the activity of sympathetic and parasympathetic nerves.

Although BMI is a measure of the relationship between the body weight and height, individuals with strong or dense bones or muscles can be mistaken as overweight or obese, and individuals with a normal BMI may actually have a percentage of high body fat. Including WC can mitigate the inadequacies of BMI and also determine the amount of fat accumulation in the abdomen [32]. In other words, WC is a health assessment index; not only does it reflect the degree of obesity in the body and the severity of visceral fat accumulation, but also it is a way to measure the risks of chronic disease and cardiovascular disease [33].

As for the relationship between shift work and HRV, one study examined middle-aged individuals (mean age 45 ± 10 years) and observed that shift work increases work pressure, which affects the sympathetic nerves [34]. Ito et al. analyzed nurses with a mean age of 33 ± 3 years and found no significant differences between their HRV-related indicators when they worked night shifts or day shifts [35]. Indeed, our study found that the differences between the HRV parameters of nurses with an overly large WC or an overly high BMI and those of nurses with normal or smaller/lower WC or BMI did not vary with shift type. In other words, the impact of an overly large WC or overly high BMI on HRV is not affected by shift type. The mean age of the nurses in this study though was lower than those in the two studies mentioned above, and fewer years working rotating shifts could explain the reduced influence of shift type.

The nurses examined in this study were younger than participants in other studies, which prevented us from determining whether long-term shift work strengthened the impact of obesity on HRV. We suggest that more large-scale studies be performed in the future and that longitudinal studies be conducted to verify the moderating effects of age or shift type on the impact of obesity on HRV in nurses.

The ANS plays a dominating role in the regulation of many functions in the body. The cardiac ANS includes the sympathetic nervous system and the parasympathetic nervous system. In the cardiovascular system, the heart rate and the contraction and relaxation of blood vessels are controlled by the cardiac ANS. The sympathetic nervous system can accelerate the heart rate, contract the blood vessels, and increase blood flow resistance, whereas the parasympathetic nervous system can lower the heart rate, relax the blood vessels, and reduce blood flow resistance. These two systems perform their functions in a mutually competing way. Therefore, the drive of the sympathetic and parasympathetic nervous systems, the baroreflex of the carotid sinus and the aortic arch, and the vasomotor tone of the surrounding blood vessels all regulate the heartbeat in the body [36]. Note that this study did not investigate the regulation process of the vasomotor tone of the surrounding blood vessels. We suggest that future studies include physical activity interventions to observe whether the parasympathetic activity in obese nurses with lower autonomic nerve activity can be improved. Furthermore, HRV reflects autonomic nerve activity levels and can serve as a crucial indicator of an individual's overall health. Abnormal HRV means poor autonomic nerve functions and may indicate poor physical and mental health [37]. Note also that we did not collect such data in this study; therefore, we suggest that future studies could also take physical and mental factors into account.

## 5. Conclusion

In conclusion, after age and shift type were controlled, this study found that nurses with an overly large WC had significantly lower HF, LF, and SDNN, while nurses with an overly high BMI had significantly lower VLF and SDNN. The age of nurses strengthens the influence of an overly large WC on HF and LF and also strengthens the influence of an overly high BMI on HF, LF, and VLF. In contrast, shift type did not moderate the impact of an overly large WC or overly high BMI on HRV.

## Figures and Tables

**Table 1:** Demographic characteristics and HRV values of participants.

Variable	All (*N* = 242)	Rotating shifts (*n* = 178)	Fixed day shift (*n* = 64)	*p* ^†^
	*n* (%)	*n* (%)	*n* (%)	
Gender				0.085
Male	8 (3.3)	8 (4.5)	0 (0)	
Female	234 (96.7)	170 (95.5)	64 (100)	
Smoking habit				0.255
None	230 (95.0)	167 (93.8)	63 (98.4)	
Occasionally	5 (2.1)	4 (2.2)	1 (1.6)	
Has quit	7 (2.9)	7 (3.9)	0 (0)	
Drinking habit				0.136
None	178 (73.6)	92 (51.7)	40 (62.5)	
Occasionally	64 (26.4)	86 (48.3)	24 (37.5)	
Hypertension				0.548
Yes	1 (0.4)	1 (0.6)	0 (0)	
No	241 (99.6)	177 (99.4)	64 (100)	
Diabetes				0.112
Yes	3 (1.2)	1 (0.6)	2 (3.1)	
No	239 (98.8)	177 (99.4)	62 (96.9)	
	Mean (SD)	Mean (SD)	Mean (SD)	
Age	28.98 (6.56)	27.09 (4.59)	34.26 (8.18)	<0.001
BMI	23.16 (4.69)	23.09 (4.44)	23.35 (5.38)	0.707
Systolic pressure	103.88 (12.67)	103.44 (11.80)	105.09 (14.86)	0.425
Diastolic pressure	65.78 (10.09)	65.78 (10.09)	65.78 (10.09)	0.855
WC	76.98 (11.17)	76.50 (10.76)	78.31 (12.24)	0.268
HR	74.75 (11.36)	74.41 (10.62)	75.71 (13.24)	0.435
HF	6.26 (1.59)	6.32 (1.46)	6.12 (1.90)	0.442
LF	6.39 (1.50)	6.42 (1.43)	6.29 (1.68)	0.541
LF/HF	1.04 (0.18)	1.03 (0.16)	1.06 (0.23)	0.259
VLF	6.57 (1.31)	6.58 (1.30)	6.54 (1.35)	0.833
TP	7.62 (1.18)	7.69 (1.12)	7.45 (1.30)	0.172
SDNN	75.59 (107.36)	75.16 (105.13)	76.80 (114.18)	0.917

^†^Categorical variables were analyzed using a chi-square test, and continuous variables were analyzed using a *t*-test. Abbreviations: BMI: body mass index; WC: waist circumference; HRV: heart rate variability; HF: high frequency; LF: low frequency; LF/HF: low- to high-frequency power ratio; VLF: very low frequency; TP: total power; SDNN: standard deviation of normal-to-normal interval.

**Table 2:** Summary table of regression coefficients of influence of WC on HRV.

Variable	HF			LF			LF/HF			VLF			TP			SDNN		
	*B*	*β*	*p*	*B*	*β*	*p*	*B*	*β*	*p*	*B*	*β*	*p*	*B*	*β*	*p*	*B*	*β*	*p*
WC-overly large^†^	-0.49	-0.14	0.026	-0.44	-0.14	0.035	0.02	0.04	0.504	-0.31	-0.11	0.091	-0.23	-0.09	0.146	-32.80	-0.14	0.029
Age	-0.04	-0.16	0.030	-0.03	-0.13	0.075	00.002	0.07	0.358	-0.02	-0.09	0.232	-0.04	-0.24	0.001	-0.76	-0.05	0.526
Shift type-fixed day shift	0.12	0.03	0.654	0.12	0.04	0.629	0.02	0.04	0.624	0.11	0.04	0.602	0.10	0.04	0.618	10.02	0.04	0.574
*R* ^2^		0.046			0.036			0.011			0.020			0.064			0.023	
*F*		1.87			2.96			0.90			1.60			5.44			1.85	
*p*		0.010			0.033			0.441			0.190			0.001			0.139	
BMI-overly high^†^	-0.35	-0.10	0.103	-0.34	-0.11	.089	0.01	0.02	0.757	-0.40	-0.15	0.023	-0.20	-0.08	0.200	-33.60	-0.15	0.020
Age	-0.04	-0.17	0.018	-0.03	-0.14	0.049	0.002	0.07	0.325	-0.02	-0.10	0.179	-0.04	-0.25	0.001	-0.99	-0.06	0.410
Shift type-fixed day shift	0.12	0.03	0.640	0.13	0.04	0.610	0.02	0.04	0.623	0.13	0.04	0.554	0.10	0.04	0.601	11.04	0.05	0.535
*R* ^2^		0.037			0.030			0.010			0.029			0.062			0.025	
*F*		3.07			2.43			0.78			2.41			5.27			2.07	
*p*		0.028			0.066			0.504			0.068			0.002			0.104	

*B* is the unstandardized regression coefficient, and *β* is the standardized regression coefficient. ^†^WC ≤ 90 cm for men and WC ≤ 80 cm for women served as the reference group; BMI < 24 kg/m^2^ served as the reference group. Abbreviations: BMI: body mass index; WC: waist circumference; HRV: heart rate variability; HF: high frequency; LF: low frequency; LF/HF: low- to high-frequency power ratio; VLF: very low frequency; TP: total power; SDNN: standard deviation of normal-to-normal interval.

**Table 3:** Moderating effects of age on impact of WC and BMI on HRV.

Variable	HF			LF			LF/HF			VLF			TP			SDNN		
	*B*	*β*	*p*	*B*	*β*	*p*	*B*	*β*	*p*	*B*	*β*	*p*	*B*	*β*	*p*	*B*	*β*	*p*
Independent variable (*X*1: overly large WC)^†^	-0.45	-0.13	0.040	-0.40	-0.12	0.051	0.02	0.04	0.541	-0.28	-0.10	0.122	-0.22	-0.09	0.179	-31.54	-0.14	0.036
Moderator (*M*: age)	-0.002	-0.01	.934	0.0004	0.002	0.985	0.001	0.03	0.694	0.01	0.03	0.709	-0.03	-0.15	0.068	0.40	0.02	0.774
Interaction term (*X*1 × *M*)	-0.08	-0.21	0.010	-0.06	-0.18	0.030	0.004	0.08	0.334	-0.05	-0.16	0.060	-0.03	-0.11	0.167	-2.04	-0.08	0.343
*R* ^2^		0.072			0.054			0.014			0.033			0.071			0.025	
*F*		6.15			4.52			1.14			2.72			6.03			2.05	
*p*		<.001			.004			.335			.045			.001			.108	
Independent variable (*X*2: overly high BMI)^†^	-0.33	-0.10	0.113	-0.33	-0.10	0.099	0.01	0.02	0.764	-0.39	-0.14	0.025	-0.19	-0.08	0.218	-32.88	-0.15	0.023
Moderator (*M*: age)	0.005	0.02	0.808	-0.0003	-0.001	0.988	-0.0003	-0.02	0.843	0.01	0.03	0.699	-0.02	-0.13	0.127	0.34	0.02	0.804
Interaction term (*X*2 × *M*)	-0.10	-0.27	0.001	-0.07	-0.19	0.023	0.01	0.16	0.054	-0.05	-0.17	0.045	-0.04	-0.16	0.052	-2.28	-0.09	0.283
*R* ^2^		0.078			0.050			0.024			0.044			0.076			0.029	
*F*		6.73			4.14			1.96			3.67			6.53			2.34	
*p*		<0.001			0.007			0.121			0.013			<0.001			0.074	

*B* is the unstandardized regression coefficient, and *β* is the standardized regression coefficient. ^†^WC ≤ 90 cm for men and WC ≤ 80 cm for women served as the reference group; BMI < 24 kg/m^2^ served as the reference group. Abbreviations: BMI: body mass index; WC: waist circumference; HRV: heart rate variability; HF: high frequency; LF: low frequency; LF/HF: low- to high-frequency power ratio; VLF: very low frequency; TP: total power; SDNN: standard deviation of normal-to-normal interval.

**Table 4:** Moderating effects of shift type on impact of WC and BMI on HRV.

Variable	HF			LF			LF/HF			VLF			TP			SDNN		
	*B*	*β*	*p*	*B*	*β*	*p*	*B*	*β*	*p*	*B*	*β*	*p*	*B*	*β*	*p*	*B*	*β*	*p*
Independent variable (*X*1: overly large WC)^†^	-0.39	-0.11	0.138	-0.33	-0.10	0.186	0.02	0.04	0.578	-0.17	-0.06	0.436	-0.21	-0.08	0.288	-29.49	-0.13	0.097
Moderator (*M*: fixed day shift)	0.02	0.01	0.935	0.09	0.03	0.747	0.03	0.06	0.447	0.18	0.06	0.437	-0.11	-0.04	0.595	9.70	0.04	0.617
Interaction term (*X*1 × *M*)	-0.51	-0.10	0.293	-0.51	-0.10	0.263	0.01	0.01	0.875	-0.56	-0.13	0.163	-0.27	-0.07	0.445	-14.55	-0.04	0.658
*R* ^2^		.032			.028			.008			.022			.023			.022	
*F*		2.62			2.30			0.63			1.78			1.85			1.78	
*p*		0.052			0.078			0.599			0.152			0.138			0.152	
Independent variable (*X*2: overly high BMI)^†^	-0.21	-0.06	0.395	-0.28	-0.09	.236	-0.01	-0.02	0.787	-0.29	-0.110	.154	-0.19	-0.08	0.316	-32.18	-0.14	0.059
Moderator (*M*: fixed day shift)	0.01	0.004	0.961	-0.02	-0.01	0.942	0.01	0.02	0.823	0.14	0.05	0.566	-0.19	-0.07	0.376	6.17	0.03	0.757
Interaction term (*X*2 × *M*)	-0.49	-0.10	0.298	-0.23	-0.05	0.608	0.06	0.10	0.303	-0.39	-0.09	0.315	-0.07	-0.02	0.851	-5.68	-0.02	0.859
*R* ^2^		.019			.015			.010			.026			.015			.023	
*F*		1.53			1.20			0.81			2.13			1.20			1.85	
*p*		0.208			0.311			0.487			0.097			0.311			0.138	

*B* is the unstandardized regression coefficient, and *β* is the standardized regression coefficient. ^†^WC ≤ 90 cm for men and WC ≤ 80 cm for women served as the reference group; BMI < 24 kg/m^2^ served as the reference group. Abbreviations: BMI: body mass index; WC: waist circumference; HRV: heart rate variability; HF: high frequency; LF: low frequency; LF/HF: low- to high-frequency power ratio; VLF: very low frequency; TP: total power; SDNN: standard deviation of normal-to-normal interval.

## Data Availability

The statistical data used to support the findings of this study are available from the corresponding author upon request.
